# Light‐based manipulation of visual processing speed during soccer‐specific training has a positive impact on visual and visuomotor abilities in professional soccer players

**DOI:** 10.1111/opo.13423

**Published:** 2024-11-20

**Authors:** Patricia Rodrigues, Jack Woodburn, Alexander John Bond, Andrew Stockman, Jesús Vera

**Affiliations:** ^1^ Sports Vision Specialist, SC Braga Braga Portugal; ^2^ Okkulo Newcastle Upon Tyne UK; ^3^ Centre for Social Justice in Sport and Society, Carnegie School of Sport Leeds Beckett University Leeds UK; ^4^ Institute of Ophthalmology University College London London UK; ^5^ CLARO (Clinical and Laboratory Applications of Research in Optometry) Research Group, Department of Optics, Faculty of Sciences University of Granada Granada Spain; ^6^ New England College of Optometry Boston Massachusetts USA

**Keywords:** athlete, lighting conditions, plasticity, sport, sports vision training, visual perception

## Abstract

This study was aimed at assessing the effects of a 6‐week intervention within a training environment that uses special lighting conditions targeted to slow down the visual processing speed of visual and visuomotor performance in professional soccer players. Twenty‐four soccer players (age = 21.8 ± 4.8 years, 50% women) from the under 18 and under 23 men's teams, and 1st Women's team of the Sunderland Association Football Club participated in this study. Participants were randomly assigned to the intervention and control groups, with the intervention group performing 2‐weekly 30‐min sessions of specific soccer tasks with specific lighting conditions using the Okkulo system (Okkulo™, okkulo.com), whereas the control group performed the same training under normal lighting conditions. The intervention group showed significant improvements in dynamic visual acuity (*p* < 0.001), recognition time (*p* = 0.002), sensory reaction time (*p* < 0.001), motor reaction time (*p* = 0.002) and peripheral identification accuracy (*p* < 0.001), whereas no significant effects were obtained for stereopsis (*p* = 0.05), peripheral identification speed (*p* = 0.17) and anticipation (*p* = 0.22). In conclusion, a 6‐week training intervention using the Okkulo system improved several visual and visuomotor skills in professional soccer players. Future studies will assess the transfer effects of using this technology to on‐field performance.

**Key points**

- The study aimed to assess the effects of the novel ‘Okkulo’ training method on professional soccer players.
- The results of this randomised controlled trial showed substantial improvements in visual and visuomotor performance for the Okkulo‐trained athletes in comparison to controls.
- This study demonstrated that a 6‐week training intervention using the Okkulo system enhanced some visual and visuomotor skills in professional soccer players.

## INTRODUCTION

Rapid and accurate visual processing is fundamental to performing well in sport, especially in those disciplines where athletes must gather information from the environment to make correct decisions and execute motor actions to interact with a ball or object. Because there is a finite delay in responding to visual stimuli, athletes must predict where a ball or object will be slightly in the future.^1^ Numerous research studies have demonstrated a superiority in visual and visuomotor abilities of athletes when compared to non‐athletes,^2^, ^3^, ^4^, ^5^ as well as a relationship between visual and visuomotor skills with different sport performance indicators.^6^

Due to the well‐documented link between visual function and sport performance, researchers from the fields of sport science and optometry have investigated the effectiveness of different sports vision training strategies on certain sports‐specific visual and cognitive abilities and in‐game performance.^7^ Within the strategies followed for vision training, programmes targeted at enhancing multiple object tracking abilities,^8^ computerised visual training devices^9^ and stroboscopic training^10^ have been demonstrated to have a positive impact on visual and visuomotor skills and sport performance. Indeed, many athletes have incorporated vision training programmes within their habitual training regimen as a way to improve athletic performance.

Positive results have been obtained using stroboscopic training in sport disciplines such as badminton,^11^ ice‐hockey,^12^ soccer^13^, ^14^ and handball,^15^ among others. Evidence from neurophysiological studies suggests that the effectiveness of this strategy relies on the delay in visual motion perception during stroboscopic eyewear,^16^ which results in a more efficient processing of the visual information in the long term,^11^, ^17^ but it is also affected by a loss of visual information. Research in the field of vision science has shown that visual motion and flicker perception are affected by light adaptation and thus can be manipulated by altering the level of illumination.^18^ The responses of both rods and cones speed up in the light and slow down in the dark, so that at lower levels of illumination, visual processing is slower. Cones, which are mainly responsible for detecting balls and objects when we fixate on them with our central vision (where rods are absent), can be approximately 50 ms slower in the dark.^18^, ^19^, ^20^

A novel sports training environment has been developed by Okkulo™ (okkulo.com) that takes advantage of the natural processes of human light adaptation by manipulating the effective light levels in which athletes train. The technology is based on training under lighting conditions that slow down visual processing, after which the athlete's visual performance and reaction times improve when they return to normal lighting levels and normal processing speeds. Okkulo mainly takes advantage of the slowing down of the cones, but processing at low light levels may also be affected by slower rods further away from central fixation.^21^, ^22^, ^23^, ^24^ To date, the scientific validity of this system for the targeted purpose is limited to unpublished data and the enthusiastic subjective reports of participants, and thus, further testing is required with well‐controlled experimental designs. The current randomised controlled trial was designed to determine the effects of a 6‐week training programme with the Okkulo system on visual and visuomotor skills in professional soccer players. The main hypothesis was that soccer players after training in the reduced light‐based environment (intervention group), where visual processing and motion perception are slowed down, would have significantly higher performance improvements on the assessments of visual and visuomotor abilities than controls when they return to normal lighting conditions.

## METHODS

### Participants

An apriori power analysis (G* Power [version 3.1.9], psychologie.hhu.de/arbeitsgruppen/allgemeine‐psychologie‐und‐arbeitspsychologie/gpower), considering a repeated‐measures analysis of variance (ANOVA) with the between‐subject factor group (control, intervention) and the within‐subject factor time (pre, post), as well as an α‐level of 0.05, power of 0.8 and effect size (*f*) of 0.28 (60% of the effect size obtained by Hülsdünker et al.^25^) projected a required sample size of 20 participants. Twenty‐four soccer players (mean age 21.8 ± 4.8 years, age range: 18–30 years, 12 men and 12 women) were recruited to participate in this study. The study was conducted with the under 18 years of age and under 23 years of age men's teams (the four players recruited from the under 18 team were 18 years old at the beginning of the intervention), and 1st Women's team of Sunderland Association Football Club (AFC) in the UK. All were screened for the following inclusion criteria: (i) visual acuity ≤0.00 log MAR in each eye with their best refractive correction, (ii) no history or presence of ocular surgery or disease based on a thorough ocular history, slit‐lamp and fundus examination, (iii) no binocular, accommodative or oculomotor dysfunction, as assessed by a visual examination performed by a board‐certified optometrist (PR) and (iv) being free of any physical limitation that could compromise the tested performance. The study protocol adhered to the Code of Ethics of the World Medical Association (Declaration of Helsinki) and was approved by the Institutional Review Board.

### Experimental design and procedure

**FIGURE 1:**
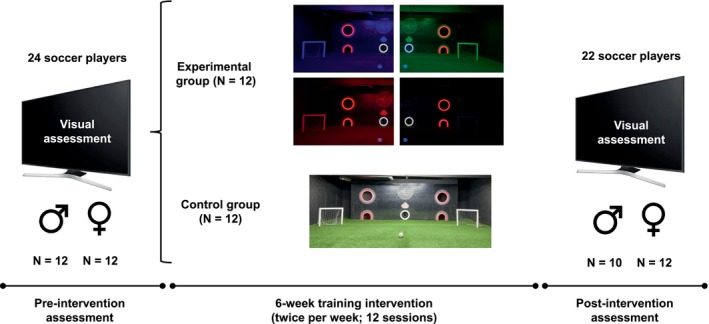
A schematic illustration of the experimental design.

This experiment was designed as a 6‐week intervention study, with visual tests being performed prior to and after the 6‐week training intervention. The pre‐ and post‐intervention assessments were identical and included eight different tests (see the subsection ‘Visual assessment’). Participants were randomly assigned to the intervention and control groups (1:1 ratio) by using a random numbers generator (graphpad.com). Players in the intervention group performed two weekly sessions (30 min per session) with the Okkulo system (Okkulo™, okkulo.com), whereas the control group performed the same training exercises using normal lighting conditions (see Figure 1 for a schematic illustration of the experimental protocol). Pre‐ and post‐intervention tests were conducted at Sunderland AFC training ground, as well as training sessions with an Okkulo system built for the club. Two players, one from each experimental group, suffered a soccer injury during the course of the study, and thus, they did not complete the investigation. A total of 22 participants (experimental group: 6 women and 5 men; control group: 6 women and 5 men) performed all training sessions and were included in the post‐intervention assessment.

### Visual assessment

For pre‐ and post‐intervention visual assessments, the COI‐Sport Vision software (OptoSolutions—COIVision, optosolutions.es) with a 55‐inch screen (Samsung Flip Pro, samsung.com) was used. This software provides assessment of visual, cognitive and sensorimotor skills, and has been used in previous investigations within the field of sports vision.^26^, ^27^ As indicated by Erickson,^28^ computer‐based evaluations permit assessment of visual, cognitive and sensorimotor skills in athletes using a standardised procedure. All visual assessments were performed with the COI‐Sport Vision software. The post‐intervention assessments were conducted between 2 and 5 days after the last training session. In the current study, the following visual and visuomotor skills were assessed:

- Dynamic visual acuity (DVA): For assessing the smallest identifiable size of a moving target, letter optotypes with 100% contrast were presented. The movement was set at an initial angular speed of 60 revolutions per minute (rpm). The computerised Wayne Rotator system integrated in the COI‐Sport Vision software was utilised. The viewing distance for this test is 6 m, and letters of different sizes appear on the panel in front of the subject. Participants were instructed to identify the moving targets (four letters and one number) of the line corresponding to visual acuity of 0.10 logMAR while the speed decreases in steps of 1 rpm per second. The maximum angular speed at which subjects can identify the five targets is recorded. Participants were previously familiarised with the procedure, and the targets were randomly changed across trials.
- Recognition time: The ability to distinguish one stimulus among a group of inputs that cause confusion. The subject, who is 5 m away from the screen, must identify four numbers appearing in the centre of the screen (0.30 logMAR), which are surrounded by moving distractors across the screen. Initially, the stimuli were presented for a duration of 100 ms, and if they were correctly identified, then the presentation time was decreased in 10 ms steps until the participants was unable to recognise the targets. Two trials were performed, and the minimum time required for recognising the targeted stimulus was considered for analyses.
- Stereopsis: The ability to integrate images acquired via each eye into a single three‐dimensional image. This test allows identification of the smallest stereoscopic disparity that the subject can distinguish, with results in seconds of arc, using the bichromatic (red‐green) binocular dissociation technique. The subject stands 3 m from the screen and has to indicate which of the five stimuli has a degree of disparity (i.e., there is only one from the five presented symbols). The disparity is reduced until obtaining the value of fine depth discrimination, with the minimum disparity displayed by the software being 10 s of arc. When the subject failed to indicate the correct stimulus, the order of the five targets corresponding to the given disparity was randomly modified, and subjects were asked again to ensure the endpoint for analysis.
- Reaction time (sensorial and motor): The ability to react to a given stimulus. In the test, two targets appear on the screen, and the athlete is asked to tap one of the targets until it changes colour. At this point, he/she has to stop pressing and tap on the other target as fast as possible. The time from the target colour change to stop pressing is computed as sensory reaction time, whereas the time required for tapping on the second target is considered as motor reaction time. Participants were instructed to use their dominant hand.
- Peripheral identification: The ability to see objects in the field of vision that were not in direct line of sight. In this test, the subject must look at the target presented in his/her line of sight (i.e., centre of the screen), and different targets appear in the periphery. The targets are circles (5 cm radius) of different colours, with the central reference circle being red and the peripheral circles being of five different colours. Participants must press the reference target as quickly as possible when the same stimulus is presented in the periphery. The results are recorded in terms of correct responses and mean reaction time from a total of 32 trials performed. The test is performed at a distance of 0.5 m between the subject and the screen. Targets are presented at different eccentricities following a randomised order, which was standardised to avoid differences across participants.
- Anticipation: This aims to assess the ability to predict the movement of objects. In this test, a moving object (a red ball of 3.5 cm radius) moves across the screen at a constant linear speed, and then suddenly disappears. The subject must touch the screen when he/she considers that the target reaches the defined area (5 cm radius) on the screen by predicting its trajectory. The goal is for the subject to predict when the ball will pass through the specified area by pressing a sensor. Ten trials were performed, with speeds and movement trajectories being randomly modified across trials, and the percentage of correct responses considered to determine anticipation performance. The allowed time error to count as a correct response regardless of the movement characteristics was 30 ms.

### Okkulo system

The Okkulo system has been designed to enhance athletes’ visual processing while performing sport‐specific tasks. This technology permits the execution of training drills under specific lighting conditions and with fluorescent materials that aim to cause a slowing down of the response of the visual system. It is based on extensive scientific evidence from laboratory studies showing the impact of reducing the light levels on the visual processing speed.^18^, ^19^, ^23^

Specifically, by varying the spectral composition and intensities of the lighting, the processing speeds of the cone and the rod photoreceptors in the human eye can be modified. The ‘Okkulo’ light of 365 nm is largely invisible to the cones and rods but causes objects, such as balls, to fluoresce, so that they can still be seen by the photoreceptors despite the otherwise very low light levels. Because of these light levels, photoreceptor processing speeds are much slower than under normal light levels by as much as 50 ms for the cones.^18^, ^19^ Rods are absent in the centre of vision, so that processing where athletes fixate depends mainly on cones. Rods may also be involved in visual processing in the periphery (around the centre). Rod vision relative to cones can be delayed by up to 50–60 ms at very low light levels,^29^ but at higher ‘mesopic’ levels at which rods and cones normally operate together, the delay can halved.^30^ Because visual processing under the training conditions is slowed down, athletes have less time to respond and so must recalibrate their visual and visuomotor responses to interact successfully with the ball or object.

In this study, four lighting conditions, corresponding to the wavelengths depicted in Table 1, were used for the intervention group. For the six‐week intervention, participants in the intervention group trained under the following lighting conditions: week 1: blue‐light, week 2: green‐light, week 3: red‐light and week 4: ‘Okkulo‐light’, while in weeks 5 and 6: red‐ and Okkulo‐light conditions were used alternatively. Participants in the control group trained during the 6‐week intervention in the named ‘normal‐lighting’ conditions (see Table 1). The illuminance levels of the different lighting conditions are shown in Table 1, which were quantified in the main areas of interest with a calibrated light meter (P‐9710‐1, Gigahertz Optik GmbH, gigahertz‐optik.com). The important lighting conditions are the Okkulo‐light and the red‐light conditions for which the lux levels are very low. The blue‐light and green‐light conditions are brighter for the cones but also much brighter for the rods.^31^

**TABLE 1:** Dominant wavelengths and illuminance levels in the principal lighting conditions used for this study. Okkulo refers to a novel sports training environment developed to take advantage of the natural processes of human light adaptation.

	Dominant wavelength (nm)	Illuminance levels in the main areas of interest (lux)		
		Outfielder player area (lux)	Player view (eye level) (lux)	Ball (outfield area) (lux)
Normal	555.5	903	38.68	64.48
Blue	450	18.47	0.53	0.77
Green	530	13.99	0.65	0.79
Red	630	0.18	0.09	0.16
‘Okkulo’	365	0.49	0.10	0.18

*Note*: All measurements of illuminance were collected at the starting position of the player for the training exercises (see the Data S1). (i) For the ‘outfielder player area’, the measurement was taken at the floor level. (ii) For the ‘player view (eye level)’, the light meter was positioned at 180 cm above the floor. (iii) The ‘Ball (outfield area)’ measurement was taken at the floor level with the ball placed 1 m from the light meter.

The Okkulo system uses artificial light and emits some light within the ultraviolet (UV) spectrum. Consequently, it must meet the obligations of UV light exposure limit values defined by The Control of Artificial Optical Radiation at Work Regulations 2010.^32^ An external assessment was conducted by UV Light Technology Limited (uv‐light.co.uk), which concluded that 3 h per day must be considered as the exposure time limit for anyone not wearing UV blocking spectacles.

### Intervention programme

Participants attended the training sessions twice per week. The sessions were supervised by a trained Okkulo staff member who provided instructions on the training exercises. Each session lasted approximately 30 min (this time is well below the 3 h of UV exposure time limit at the level used in the system), and the lighting conditions used for each training session have been explained in the Okkulo system subsection. The athletes were required to use only a set group of drills, which were designed to be fitting for all positions on the pitch. Each drill was simple and was to be completed individually with the Okkulo coach. All sessions would use the ball machine for at least part of the session. During the intervention programme, players followed their habitual schedule, including their daily training routine and matches. A detailed description with schematic illustrations of the exercises included in the training sessions can be found in the Data S1.

### Statistical analysis

Descriptive data are presented as means ± standard deviation. The normal distribution of the data was checked with the Shapiro–Wilk test and the homogeneity of variances with the Levene's test. Separate repeated measures analysis of variance (ANOVA) with the group (control vs. intervention) as a between‐participants factor and time (pre‐ and post‐intervention) as the only within‐participants factor was performed for each dependent variable. Non‐parametric tests were used if a normal distribution was violated, while in case of non‐sphericity, the degrees of freedom were corrected based on the Greenhouse–Geisser method. When obtaining statistically significant interaction effects, pairwise comparisons for paired and independent samples were performed with the Wilcoxon signed‐rank and Mann–Whitney *U* tests, respectively. Both tests are robust to outliers and do not require the homogeneity of variances assumption to be met.^33^ The magnitude of the changes was reported by the rank‐biserial correlation coefficient (*r*_*b*_), and the Holm–Bonferroni correction was applied for controlling the effects of multiple testing. Statistical significance was set at an alpha level of 0.05.

## RESULTS

### Control analyses

To check for possible differences between groups, independent *t*‐tests were performed for athletes characteristics (age and years of training) and the visual and visuomotor parameters assessed (before training) in this study. This analysis showed that there were no statistically significant differences for any of these variables between groups (all *p* > 0.05).

### Main analyses

Table 2 shows the descriptive and statistics values for the different dependent variables assessed in this study.

**TABLE 2:** Descriptive and statistics values for the visual and visuomotor skills assessed in this study. Data from both groups (control and experimental) and time of measurement (pre‐ and post‐training) are depicted.

Dependent variables	Group	Descriptive (mean ± standard deviation)		Statistics (*p*‐value; effect size [ƞ^2^_*p*_])		
		Pre‐training	Post‐training	Time	Group	Time × Group
Dynamic visual acuity (rpm)	Control	50.7 ± 7.6	49.7 ± 8.2	***p* = 0.009** **ƞ** ^**2**^_***p***_ **= 0.29**	***p* = 0.008** **ƞ** ^**2**^_***p***_ **= 0.30**	***p* < 0.001** **ƞ** ^**2**^_***p***_ **= 0.49**
	Experimental	54.8 ± 3.2	59.6 ± 0.9			
Recognition time (ms)	Control	68.2 ± 38.2	83.6 ± 20.6	*p* = 0.12 ƞ^2^_*p*_ = 0.12	*p* = 0.07 ƞ^2^_*p*_ = 0.16	***p* < 0.001** **ƞ** ^**2**^_***p***_ **= 0.51**
	Experimental	70.9 ± 29.8	38.2 ± 22.1			
Stereopsis (seconds of arc)	Control	32.7 ± 18.5	43.6 ± 36.4	*p* = 0.94 ƞ^2^_*p*_ = 0.01	*p* = 0.14 ƞ^2^_*p*_ = 0.10	*p* = 0.05 ƞ^2^_*p*_ = 0.17
	Experimental	31.8 ± 18.3	20.0 ± 10.0			
Sensory reaction time (ms)	Control	436.2 ± 40.3	417.9 ± 29.0	***p* < 0.001** **ƞ** ^**2**^_***p***_ **= 0.58**	*p* = 0.14 ƞ^2^_*p*_ = 0.10	***p* = 0.004** **ƞ** ^**2**^_***p***_ **= 0.34**
	Experimental	439.7 ± 59.9	364.1 ± 29.9			
Motor reaction time (ms)	Control	255.5 ± 45.5	296.7 ± 70.5	*p* = 0.16 ƞ^2^_*p*_ = 0.10	***p* = 0.004** **ƞ** ^**2**^_***p***_ **= 0.034**	***p* < 0.001** **ƞ** ^**2**^_***p***_ **= 0.52**
	Experimental	255.8 ± 49.3	177.3 ± 33.9			
Peripheral identification (*N*)	Control	30.4 ± 1.3	31.1 ± 0.9	***p* < 0.001** **ƞ** ^**2**^_***p***_ **= 0.46**	*p* = 0.10 ƞ^2^_*p*_ = 0.13	***p* = 0.02** **ƞ** ^**2**^_***p***_ **= 0.25**
	Experimental	28.4 ± 2.5	31.5 ± 1.0			
Peripheral identification (ms)	Control	1.62 ± 0.30	1.55 ± 0.47	***p* = 0.03** **ƞ** ^**2**^_***p***_ **= 0.23**	*p* = 0.14 ƞ^2^_*p*_ = 0.11	*p* = 0.17 ƞ^2^_*p*_ = 0.10
	Experimental	1.54 ± 0.28	1.26 ± 0.16			
Anticipation (%)	Control	17.4 ± 20.1	19.5 ± 20.9	*p* = 0.14 ƞ^2^_*p*_ = 0.11	*p* = 0.15 ƞ^2^_*p*_ = 0.10	*p* = 0.22 ƞ^2^_*p*_ = 0.08
	Experimental	19.3 ± 27.2	41.0 ± 30.1			

*Note*: Statistically significant effects are in bold (*p* < 0.05).

Abbreviations: ms, milliseconds; *N*, number of correct responses; rpm, revolutions per minute.

**FIGURE 2:**
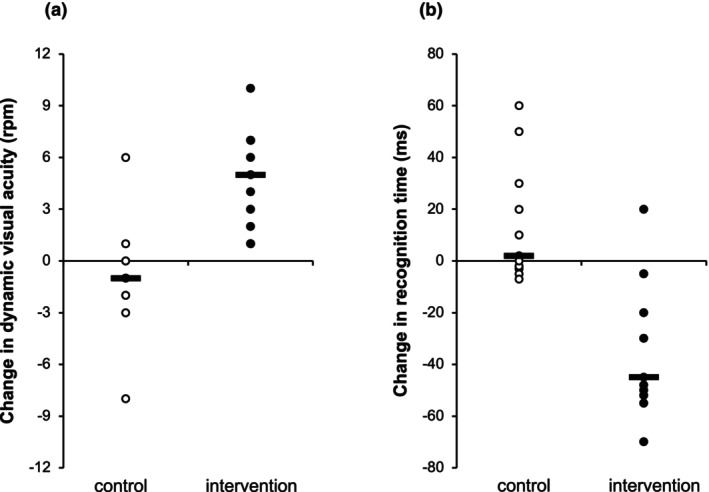
Changes in dynamic visual acuity (a) and recognition time (b) as a function of the 6‐week training programme (post‐intervention–pre‐intervention). Individual data points with the corresponding median values are displayed for the control (empty circles) and intervention (filled circles) groups. In panel (a), values higher than 0 indicate a better performance, whereas in panel (b), values lower than 0 represent a better performance. rpm, revolutions per minute.

Analysis of DVA showed statistically significant differences for the main effects of time and group (*p* = 0.009, ƞ^2^_*p*_ = 0.29 and *p* = 0.008, ƞ^2^_*p*_ = 0.30, respectively) and the interaction of time × group (*p* < 0.001, ƞ^2^_*p*_ = 0.49). Specifically, the experimental group improved DVA (corrected *p* < 0.001, *r*_*b*_ = 0.89), whereas no changes were observed for the control group (corrected *p* = 0.30) (Figure 2a). For recognition time, statistically significant differences were found for the interaction time × group (*p* < 0.001, ƞ^2^_*p*_ = 0.51). Post‐hoc analyses revealed an improvement in recognition time for the experimental group (corrected *p* = 0.002, *r*_*b*_ = 0.79), but not for the control group (corrected *p* = 0.16; Figure 2b). When considering stereopsis, there were no statistically significant differences for the main or interaction effects (time: *p* = 0.94, ƞ^2^_*p*_ = 0.01; group: *p* = 0.14, ƞ^2^_*p*_ = 0.10; and time × group: *p* = 0.05, ƞ^2^_*p*_ = 0.17).

**FIGURE 3:**
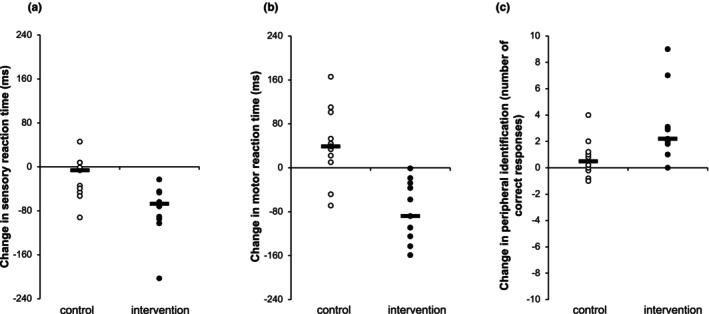
Changes in sensory reaction time (a), motor reaction time (b) and peripheral identification accuracy (c) as a function of the 6‐week training programme (post‐intervention–pre‐intervention). Individual data points with the corresponding median values are displayed for the control (empty circles) and intervention (filled circles) groups. In panels (a, b), values lower than 0 indicate a better performance, whereas in panel (c), values higher than 0 represent a better performance.

We also tested the effects of training with the Okkulo system on sensory and motor reaction time. The analysis of sensory reaction time revealed statistically significant differences for the main effect of time (*p* < 0.001, ƞ^2^_*p*_ = 0.58) and the interaction time × group (*p* = 0.004, ƞ^2^_*p*_ = 0.34). A significant improvement in sensory reaction time was found for the experimental group (corrected *p* < 0.001, *r*_*b*_ = 0.89), whereas no significant changes were observed for the control group (corrected *p* = 0.51; Figure 3a). For motor reaction time, the main effect of group (*p* = 0.004, ƞ^2^_*p*_ = 0.34) and the interaction effect of time × group (*p* < 0.001, ƞ^2^_*p*_ = 0.58) yielded statistical significance. Post‐hoc analyses showed a reduction (improvement) of motor reaction time for the experimental group (corrected *p* = 0.002, *r*_*b*_ = 0.89; Figure 3b).

For peripheral identification, the number of correct responses and speed were analysed. The analysis of correct responses evidenced a significant effect for the main factor of time (*p* < 0.001, ƞ^2^_*p*_ = 0.46), as well as the interaction time × group (*p* = 0.02, ƞ^2^_*p*_ = 0.25). Specifically, a better peripheral identification performance (more correct responses) was obtained for the experimental group (corrected *p* < 0.001, *r*_*b*_ = 0.88), whereas no significant differences were found for the control group (corrected *p* = 0.64; Figure 3c). For its part, peripheral identification speed only revealed statistical significance for the main effect of time (*p* = 0.03, ƞ^2^_*p*_ = 0.23). Lastly, the effects of training with the Okkulo system on anticipation were examined, but both main effects and the interaction were far from reaching statistical significance (*p* > 0.14).

## DISCUSSION

This study was designed to assess the effectiveness of a 6‐week training programme with the Okkulo system on visual and visuomotor skills in professional soccer players. The data show a significant improvement in several of the skills tested in soccer players training with the Okkulo system in comparison with active controls. Specifically, the use of the Okkulo system was associated with improvements in DVA, recognition time, reaction time and peripheral identification accuracy. However, no significant effects were found for stereopsis, peripheral identification speed and anticipation. Taken together, the implementation of a 6‐week training programme with the Okkulo system seems to affect some visual and visuomotor skills positively.

An 8.4% improvement in DVA was found after performing the 6‐week training intervention with the Okkulo system. This visual skill is of great relevance in soccer, since players need to perceive multiple moving stimuli (i.e., ball, opponents and teammates) during the game. Indeed, athletes, in general, have demonstrated superior DVA to non‐athletes,^34^, ^35^ and this visual skill has been associated with the ability to track multiple moving objects and in‐game performance.^36^, ^37^ Due to the fundamental role DVA plays in sport performance, researchers have assessed the effectiveness of different vision training methods on DVA. For example, there are results showing that stroboscopic vision training and interventions based on oculomotor exercises led to better DVA.^38^, ^39^ The present results show that a 6‐week training intervention with the Okkulo system was also effective in enhancing DVA in professional soccer players. Future studies are required to investigate the transferability of these improvements to in‐game performance.

The time required for processing visual stimuli plays a pivotal role in sports performance, since faster processing of visual information facilitates making the right decision and executing a successful sports action.^2^ In the current study, a significant effect (improvements ranging from 10.4% to 59.9%) was found for the training intervention on the time required to react to a static stimulus (i.e., sensory and motor reaction times), the time required to detect a moving target among confusion‐inducing stimuli (i.e., recognition time) and the ability to accurately identify stimuli in the visual periphery (i.e., peripheral identification). Evidence from stroboscopic and sports vision training protocols, using optometric tests and neurophysiological indices to assess visual processing speed, supports the positive impact of these interventions on different metrics of visuomotor reaction time in sport disciplines such as badminton, ice hockey, football, volleyball and handball.^14^, ^15^, ^25^, ^40^, ^41^ As shown for other vision training procedures, these findings provide preliminary evidence regarding the effectiveness of 6‐week soccer‐specific training under Okkulo lighting conditions on measures associated with the accuracy and speed of visuomotor reactions in soccer players.

### A plausible physiological explanation

As previously explained, the speed of visual processing of both cone and rod‐mediated visual pathways depends on light levels. Thus, the effectiveness of Okkulo is speculated to be largely due to the natural processes of light adaptation by which photoreceptors change their temporal integration times in response to changing light levels. This stops them saturating as the light level increases but has the added benefit of speeding up their responses.^42^ The Okkulo system uses lighting conditions designed to slow down visual processing speed, with the result that the visual system must respond more quickly if the athlete aims to perform a sporting task successfully. This ability to respond more quickly may translate to an advantage when the athletes return to the normally lit scenarios, since they seem to have more time to respond. This study was the first to assess the effects on athletes' visual performance after conducting soccer‐specific training in the Okkulo system.

Stroboscopic training also improves the processing of visual information while performing sport‐specific activities.^10^ Stroboscopic training has been demonstrated to be effective in enhancing the athlete's visual system and ultimately improving their performance on the field.^11^, ^17^, ^25^, ^41^, ^43^ Researchers in the field of stroboscopic training argue that the more limited visual information under stroboscopic conditions forces athletes to create new mechanisms for being able to react and make decision more quickly.^43^ The effectiveness of the Okkulo system does not limit information per se, but instead slows it down, which is a different and more natural strategy, since low light levels are experienced in the natural environment, whereas stroboscopic flicker is not.

### Limitations and future research

As with any discipline, the practice of sports vision training must be based on the best available scientific evidence. The current study incorporates insights into the effectiveness of a novel sports vision training strategy on visual and visuomotor skills in professional soccer. However, this is the first scientific study using this technology, and therefore, the findings must be interpreted cautiously in this regard and corroborated in future investigations. As explained by Laby and Appelbaum,^6^ the main goal of sports vision training is that better visual abilities underlie improved athletic performance. Therefore, the development of well‐controlled and sufficiently powered studies using the Okkulo system for testing the effects of this sports vision training strategy on in‐game performance is required. Also, the inclusion of placebo control and interventions where the light conditions are manipulated (i.e., using lights of different spectral composition and intensity) should be investigated to determine if these conditions cause different training results. One of the strengths of the current study is the inclusion of professional soccer players, which is commonly challenging due to the logistic hurdles and limited time availability. However, the external validity of the current findings to other sport disciplines or levels of sport performance should be tested in future investigations.^5^ The observed effects may be particularly relevant for sports where rapid and accurate processing of visual information is crucial for successful performance (e.g., racquet sports, volleyball, basketball or boxing). Additionally, this training strategy could be beneficial for skill acquisition in non‐professional players, as it may help lay a strong foundation for athletic development.

The incorporation of sports vision training procedures is gaining interest, with athletes, clubs and federations seeing the positive effects of incorporating these approaches for sports performance.^7^ Due to the growing interest of sports vision training, new technologies and approaches are continually being developed to enhance the internal and external validity of sports vision training applications.^6^ As indicated by Hadlow et al.,^44^ effective sports vision training approaches should incorporate sport‐specific tasks and be logistically feasible and tolerable for athletes who generally have considerable time constraints. In this regard, the Okkulo system allows the integration of vision training into an athlete's habitual training routine. The present data show that training under specific lighting conditions targeted to slow down visual processing is a valid alternative to enhance the athlete's visual perception and reaction. While this technology may appear to be related to stroboscopic training, which is also targeted at enhancing the processing speed in the long term by the manipulation of visual perception with stroboscopic eyewear,^16^ its mechanisms of action, which use the natural processes of light adaptation, may be different. Nevertheless, these sports vision training approaches open new avenues for enhancing athletes’ visual and visuomotor skills by adaptations in the visual system.

## CONCLUSIONS

The findings of the current study show that a 6‐week training intervention using the Okkulo system, which is targeted to slow down visual processing speed by the use of specific lighting‐conditions, has a positive effect on several visual and visuomotor skills (i.e., DVA, recognition time, reaction time and peripheral identification accuracy) in professional soccer players. This study provides a foundation for future research endeavours, including the transfer effects of sports vision training with the Okkulo system to on‐field performance. Future studies using placebo control are also required to investigate further the effectiveness of this novel vision training approach on different sport disciplines and levels of sport performance, as well as assess the retention effects of this training strategy.

## AUTHOR CONTRIBUTIONS

**Patricia Rodrigues:** Conceptualization (equal); data curation (equal); methodology (equal); resources (equal); writing – original draft (equal). **Jack Woodburn:** Conceptualization (equal); project administration (equal); resources (equal); writing – review and editing (equal). **Alexander John Bond:** Data curation (equal); methodology (equal); writing – review and editing (equal). **Andrew Stockman:** Conceptualization (equal); investigation (equal); methodology (equal); writing – review and editing (equal). **Jesús Vera:** Conceptualization (equal); data curation (equal); formal analysis (equal); visualization (equal); writing – original draft (equal); writing – review and editing (equal).

## FUNDING INFORMATION

There is no funding associated with the work featured in this article.

## CONFLICT OF INTEREST STATEMENT

Jesús Vera and Alexander Bond declare no conflicts of interest. Patricia Rodrigues had flights and accommodation paid for by Okkulo Ltd during testing. Jack Woodburn is a full‐time employee of Okkulo Ltd. Andrew Stockman is a shareholder of Okkulo Ltd. The authors alone are responsible for the content and writing of the paper.

## Supporting information


Data S1:

## Data Availability

The data of this study are available from the corresponding author, upon reasonable request.
