# Supplementary material for: Light‐based manipulation of visual processing speed during soccer‐specific training has a positive impact on visual and visuomotor abilities in professional soccer players
Source: Ophthalmic Physiol Opt. 2024 Nov 20;45(2):504–13. doi: 10.1111/opo.13423 (PMC11823300; doi:10.1111/opo.13423)
Supplement: Supplementary file 1 — Data S1: [file OPO-45-504-s001.docx]

Supplementary information – a description of the training drills

The training sessions were supervised by a qualified Okkulo staff member, who provided participants with instructions on the drills to be completed. The control group, however, did not receive any visual training during the study period.

The athletes were required to perform a set of drills specifically designed to suit all positions on the pitch. Each drill was simple and was conducted individually with the Okkulo coach. Every session incorporated the use of a ball machine (BOLA; Bristol, UK) for part of the training to ensure a consistent ball speed. The figures below illustrate how the sessions were structured, along with a description of how each drill was carried out.


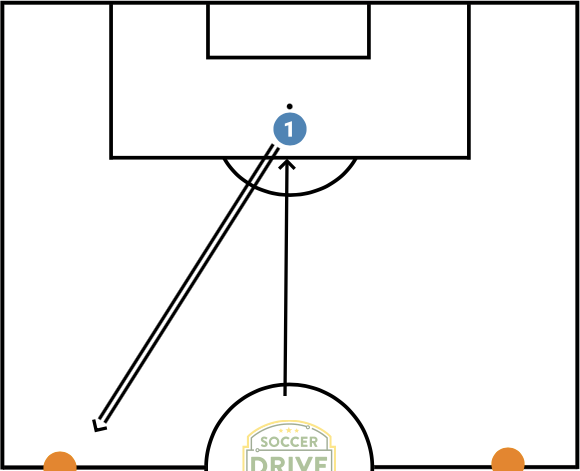


Ball Cannon

**Figure S1**. **Diagram showing one of the training exercises:** In this drill, the ball is fired from a ball cannon toward the player's feet (blue circle) at a speed of 50mph. The player must control the ball and then pass it back through the illuminated circle (one of the two orange circles).


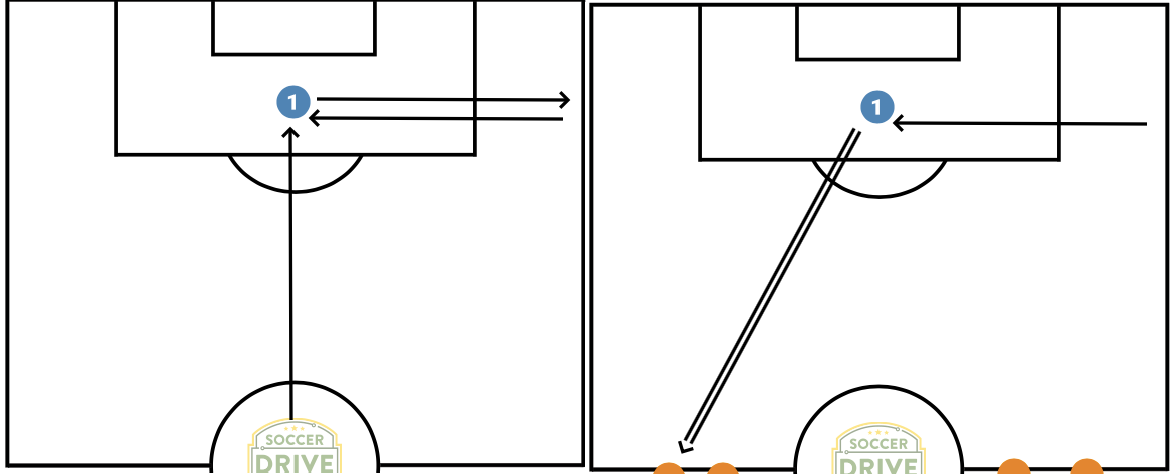


Ball Cannon

Ball Cannon

**Figure S2**: **Diagram showing one of the training exercises:** In this exercise, the ball is fired from a ball cannon toward the player's feet (blue circle) at a speed of 50mph. The player must control the ball, pass it off one of the side walls, control it again, and then pass it through the illuminated circle (one of the four orange circles).


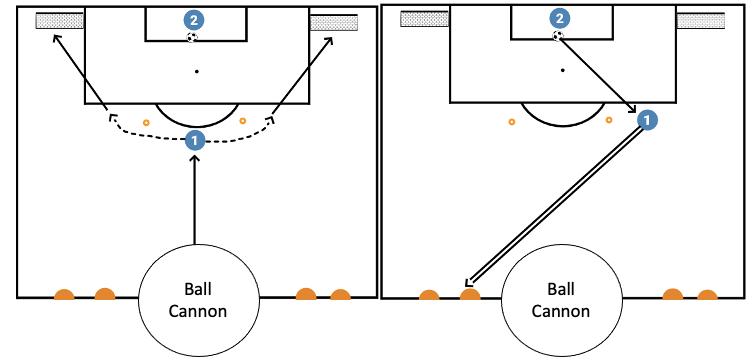


**Figure S3**. **Diagram showing one of the training exercises:** In this exercise, the ball is fired from a ball cannon toward the player's feet (blue circle, number 1) at speeds ranging from 40 to 60mph. The player must control the ball, dribbled around the cones, and passed into the goal indicated by the Okkulo coach via an audio cue (left panel). Then, the player receives a pass from the coach (blue circle, number 2) and shot the ball through the illuminated circle (one of the four orange circles, right panel).
